# Antimicrobial and antioxidant flavonoids from the leaves of *Oncoba spinosa* Forssk. (Salicaceae)

**DOI:** 10.1186/s12906-015-0660-1

**Published:** 2015-04-28

**Authors:** Marie Geneviève Djouossi, Jean-de-Dieu Tamokou, David Ngnokam, Jules-Roger Kuiate, Leon Azefack Tapondjou, Dominique Harakat, Laurence Voutquenne-Nazabadioko

**Affiliations:** Chemistry Department, University of Dschang, PO Box 67, Dschang, Cameroon; Laboratory of Microbiology and Antimicrobial Substances, Biochemistry Department, Faculty of Science, University of Dschang, PO Box 67, Dschang, Cameroon; Service Commun d’Analyse, Institut de Chimie Moléculaire de Reims (ICMR), CNRS UMR 7312, Bat. 18 BP 1039, 51687 Reims cedex 2, France; Groupe Isolement et Structure, Institut de Chimie Moléculaire de Reims (ICMR), CNRS UMR 7312, Bat. 18 BP 1039, 51687 Reims cedex 2, France

**Keywords:** *Oncoba spinosa*, Salicaceae, Flavonoids, Antibacterial, Antifungal, Antioxidant

## Abstract

**Background:**

Naturally occurring flavonoids have been reported to possess various pharmacological properties. The aim of this study was to evaluate the antimicrobial and antioxidant activities of the MeOH extract and flavonoids from the leaves of *Oncoba spinosa*, a plant used for the treatment of syphilis, wounds and sexual impotence.

**Methods:**

The plant extract was prepared by maceration in methanol and sequentially fractionated by column chromatography. The structures of isolated compounds were elucidated on the basis of spectral studies and comparison with published data. The MeOH extract and its isolated compounds were evaluated for their antibacterial and antifungal activities by broth microdilution method. The 1,1-diphenyl-2-picrylhydrazyl (DPPH) and trolox equivalent antioxidant capacity (TEAC) assays were used to detect the antioxidant activity. The samples were tested spectrophotometrically for their hemolytic properties against human red blood cells.

**Results:**

The fractionation of the MeOH extract afforded five known flavonoids including kaempferol (**1**), quercetin (**2**), apigenin-7-O-β-D-glucuronopyranoside (**3**), quercetin 3-O-β-D-galactopyranoside (**4**) and quercetin 3-O-α-L-rhamnopyranosyl (1 → 6) β-D-glucopyranoside (**5**). The MeOH extract displayed weak to moderate antimicrobial activities (MIC = 256–2048 μg/ml). Quercetin 3-O-α-L-rhamnopyranosyl (1 → 6) β-D-glucopyranoside (**5**) and quercetin (**2**) were respectively the most active compounds against bacteria (MIC = 8–64 μg/ml) and fungi (MIC = 64 – 128 μg/ml). These tested samples also showed high radical-scavenging activities (EC_50_ = 5.08 – 70.56 μg/ml) and gallic acid equivalent antioxidant capacities (TEAC = 53.76 – 89.86 μg/ml) when compared with vitamin C (EC_50_ = 4.72 μg/ml). The MeOH extract and compounds **2**–**5** were non-toxic to human red blood cells indicating their high selectivity to be used as antimicrobial and antioxidant drugs.

**Conclusion:**

The MeOH extract of *O. spinosa* as well as compounds **2 – 5** could be a potential source of natural antimicrobial and antioxidant products.

## Background

Infectious diseases are among the main cause of morbidity and mortality worldwide, with HIV, tuberculosis and malaria being the most involved. Despite the progress made in the understanding of microorganisms and their control in industrialized nations, incidents due to drug resistant microorganisms and the emergence of hitherto unknown disease-causing microbes, pose enormous public health concerns [1]. Furthermore, the development of synthetic drugs has slowed down as a result of drug resistance [2]. Consequently, this has created a new renewed interest in the search for new drugs in order to combat resistance. The need for new, effective and affordable drugs to treat microbial diseases in the developing world is one of the major issues facing global health today. Plants have been used as a source of new medicinal compounds throughout history and continue to serve as the basis for many of the pharmaceuticals used today [3]. In recent decades, many studies have been carried out on different plant species to discover compounds of possible interest for medicinal application against oxidative stress, fungal and bacterial infections. Among these studies, several have focused on the biological and phytochemical properties of different species of the family Flacourtiaceae [4-6].

*Oncoba spinosa* Forssk. belonging to the family Flacourtiaceae (Salicaceae *sensu lato*) is a small tree of about 13 m high which grows under conditions of higher rainfall, of deciduous, secondary and fringing forest from Senegal to West Cameroon, and widely distributed in tropical Africa and Arabia [7]. The plant is traditionally reputed for its medicinal potential particularly in southwest of Nigeria for the treatment of diabetes and cancer [6]. In many African countries, the leaves and roots are used for urethral discharges, infertility [8], epilepsy [9], dysentery and bladder conditions [10]. The α-glucosidase inhibitory, radical scavenging and cytotoxicity activities of the aqueous and chloroform extracts of the leaves of *O. spinosa* were reported [6]. The methanol extract of the fruits of this plant collected in Yemen demonstrated antimicrobial, anticancer and antioxidant activities [5]. Previous phytochemical studies on the genus *Oncoba* afforded three tetracyclic triterpenes from the species *O. mannii* [4]. Phytochemical screening of *O. spinosa* leaves revealed the presence of anthraquinones, alkaloids, phenols, sterols, tannins, carbohydrates and flavonoids [6].

In our continuous effort to search for novel antimicrobial/antioxidant agents from Cameroonian medicinal plants used traditionally to treat human microbial infections and oxidative related diseases [11-14], we investigated phytochemically and biologically the methanol extract of the leaves of *O. spinosa* and isolated five known compounds. They included kaempferol (**1**) [15], quercetin (**2**) [16], apigenin-7-O-β-D-glucuronopyranoside (**3**) [17,18], quercetin 3-O-β-D-galactopyranoside (**4**) [16] and quercetin 3-O-α-L-rhamnopyranosyl (1 → 6) β-D-glucopyranoside (**5**) [19]. The *in vitro* antibacterial, antifungal and antioxidant activities of the MeOH extract and compounds **2**–**5** were evaluated.

## Methods

### Experimental

Melting points were recorded with a Reichert microscope and are uncorrected. ^1^H NMR (500 MHz) and ^13^C NMR (125 MHz) were recorded at room temperature in CD_3_OD or (CD_3_)_2_SO, on a Bruker Avance DRX-500 spectrometer. Chemical shifts (δ) are reported in parts per million (ppm) with the solvent signals as reference relative to TMS (δ = 0) as internal standard, while the coupling constants (*J* values) are given in Hertz (Hz). COSY, ROESY, TOCSY, HSQC and HMBC experiments were recorded with gradient enhancements using sine shape gradient pulses. The IR spectra were recorded with a Shimadzu FT-IR-8400S spectrophotometer. ESI-MS experiments were performed using a Micromass Q-TOF micro instrument (Manchester, UK) with an electrospray source. Column chromatography was run on Merck silica gel 60 (70–230 mesh) and gel permeation on Sephadex LH-20 while TLC was carried out on silica gel GF_254_ pre-coated plates with detection accomplished by spraying with 50% H_2_SO_4_ followed by heating at 100°C, or by visualizing with an UV lamp at 254 and 365 nm.

### Plant material

The leaves of *O. spinosa* Forssk. were collected at Dschang, West Region, Cameroon, in May 2007. Authentication was done at the Cameroon National Herbarium, Yaoundé, where the voucher specimen (No. 21975 HNC) is deposited.

### Extraction and isolation

The air-dried and powdered leaves of *O. spinosa* (2 kg) were extracted by percolation in methanol for 3 days at room temperature. Evaporation of solvent under reduced pressure yielded 40 g of extract. Part of the MeOH extract (35 g) was subjected to column chromatography (silica gel 60, 70–230 mesh) and eluted with hexane followed by hexane-EtOAc gradient. Sixty fractions of 200 ml each were collected and combined on the basis of TLC analysis to afford four major fractions: A (9 g; hexane-EtOAc 100:0 and 9:1), B (8.5 g, hexane-EtOAc 4:1 and 7:3), C (11 g, hexane-EtOAc 1:1 and 0:100) and D (6.7 g, EtOAc-MeOH 19:1 and 9:1). Fraction A, mainly oil, was not further investigated in this work. Fraction B was further purified on silica gel column chromatography eluted with hexane-EtOAc 17:3, 4:1 and 3:1, respectively, to afford 30 sub-fractions (B_1_ and B_2_). Sub-fraction B_2_ (17–30) was purified through Sephadex LH-20 column chromatography eluted with CH_2_Cl_2_-MeOH 1:1 to yield kaempferol (**1**) (2.5 mg), quercetin (**2**) (5 mg) and apigenin-7-O-β-D-glucuronopyranoside (**3**) (11 mg). Silica gel column chromatography of fraction C, eluted with EtOAc-MeOH-H_2_O 9:0.5:0.5 yielded apigenin-7-O-β-D-glucuronopyranoside (**3**) (25 mg) and quercetin 3-O-β-D-galactopyranoside (**4**) (33 mg). Fraction D was purified through Sephadex LH-20 gel permeation eluted with MeOH to give 25 sub-fractions (10 ml each). Re-crystallization of these sub-fractions yielded quercetin 3-O-β-D-galactopyranoside (**4**) (6 mg), quercetin 3-O-α-L-rhamnopyranosyl (1 → 6) β-D-glucopyranoside (**5**) (24.5 mg) and complex mixtures.

- Kaempferol (**1**): yellow crystals from hexane-EtOAc; m.p. 275–277°C; C_15_H_10_O_6_.
- Quercetin (**2**): yellow needles from hexane-EtOAc; m.p. > 300°C; C_15_H_10_O_7_.
- Apigenin-7-O-β-D-glucuronopyranoside (**3**): yellow powder from EtOAc; m.p. > 300°C; C_21_H_18_O_11_.
- Quercetin 3-O-β-D-galactopyranoside (**4**): yellow needles from EtOAc-MeOH; m.p. 230–232°C; C_21_H_20_O_12_.
- Quercetin 3-O-α-L-rhamnopyranosyl (1 → 6) β-D-glucopyranoside (**5**): yellow powder from EtOAc-MeOH, m.p. 213–216°C; C_27_H_30_O_16_.

### Antimicrobial assay

#### Bacterial and fungal strains

The studied microorganisms were both reference (from the American Type Culture Collection) and clinical (from Pasteur Institute Paris, France) strains of *Enterobacter aerogenes, Escherichia coli, Klebsiella pneumoniae, Candida albicans,* and *Cryptococcus neoformans*. Also, included were two clinical isolates of *Candida parapsilosis* and *Staphylococcus aureus* collected from Pasteur Centre (Yaoundé-Cameroon). The bacterial and fungal species were grown at 37°C and maintained on nutrient agar (NA, Conda, Madrid, Spain) and Sabouraud Dextrose Agar (SDA, Conda) slants respectively.

### Preparation of microbial inoculum

The inocula of yeasts and bacteria were prepared from overnight cultures by picking numerous colonies and suspending them in sterile saline (NaCl) solution (0.90%). Absorbance was read at 530 nm for yeasts or at 600 nm for bacteria and adjusted with the saline solution to match that of a 0.50 McFarland standard solution. From the prepared microbial solutions, other dilutions with saline solution were prepared to give a final concentration of 10^6^ yeast cells/ml and 10^6^ CFU/ml for bacteria [14,20].

### Antimicrobial assay

The antimicrobial activity was investigated by determining the minimum inhibitory concentrations (MICs), minimum bactericidal concentrations (MBCs) and minimum fungicidal concentrations (MFCs).

MICs were determined by broth micro dilution [12,21]. Stock solutions of the tested samples were prepared in 10% v/v aqueous dimethylsulfoxide (DMSO) solution (Fisher chemicals, Strasbourg, France) at concentration of 4096 μg/ml. This was two-fold serially diluted in Mueller-Hinton Broth (MHB) for bacteria and Sabouraud Dextrose Broth (SDB) for fungi to obtain a concentration range of 2048 to 0.25 μg/ml. For every experiment, a sterility check (10% aqueous DMSO and medium), negative control (10% aqueous DMSO, medium and inoculum) and positive control (10% aqueous DMSO, medium, inoculum and water-soluble antibiotics) were included. One hundred microliters of each concentration was introduced into a well (96-wells microplate) containing 90 μl of SDB or MHB and 10 μl of inoculum was added to obtain a final concentration range of 4096 to 0.125 μg/ml. The plates were covered with a sterile lid, and incubated on the shaker at 37°C for 24 h (bacteria) and 48 h (yeasts). MICs were assessed visually after the corresponding incubation period and were taken as the lowest sample concentration at which there was no growth or virtually no growth. The assay was repeated thrice.

For the minimum microbicidal concentration (MMC) determination, 10 μl aliquots from each well that showed no growth of microorganism were plated on Mueller-Hinton Agar or Sabouraud Dextrose Agar and incubated at 37°C for 24 h (bacteria) and 48 h (yeasts). The lowest concentration that yielded no growth after the sub-culturing was taken as the MBCs or MFCs. Chloramphenicol (Sigma-Aldrich, Steinheim, Germany) for bacteria and nystatin (Sigma-Aldrich, Steinheim, Germany) for yeasts were used as positive controls.

### Antioxidant assay

#### DPPH free radical scavenging assay

The free radical scavenging activity of the MeOH extract as well as some of its isolated compounds was evaluated according to described methods [22,23] with slight modifications. Briefly, the test samples, prior dissolved in DMSO (SIGMA) beforehand, were mixed with a 20 mg/l 2,2-diphenyl-1-picryl-hydrazyl (DPPH) methanol solution, to give final concentrations of 10, 50, 100, 500 and 1000 μg/ml. After 30 min at room temperature, the absorbance values were measured at 517 nm and converted into percentage of antioxidant activity. L-ascorbic acid was used as a standard control. The percentage of decolouration of DPPH (%) was calculated as follows:

$$ \%\ \mathrm{decolouration}\ \mathrm{of}\ \mathrm{DPPH}=\frac{\left(\mathrm{Absorbance}\ \mathrm{of}\ \mathrm{control}\ \hbox{-}\ \mathrm{Absorbance}\ \mathrm{of}\ \mathrm{test}\ \mathrm{sample}\Big) \times 100\right)}{\mathrm{Absorbance}\ \mathrm{of}\ \mathrm{control}} $$

The percentage of decolouration of DPPH (%) was plotted against the test sample. Also, the percentage of decolouration of DPPH was converted in probits. The probit values were plotted against the logarithmic values of concentrations of the test samples and a linear regression curve was established in order to calculate the EC_50_ (μg/ml), which is the amount of sample necessary to decrease by 50% the absorbance of DPPH [11]. All the analyses were carried out in triplicate.

### Trolox equivalent antioxidant capacity (TEAC) assay.

The TEAC test was done as previously described [24] with slight modifications. In a quartz cuvette, to 950 μl acetate buffer (pH = 5.0, 100 mM), the following were added: 20 μl laccase (1 mM stock solution), 20 μl test sample, 10 μl ABTS (2,2′-azinobis(3-ethylbenzothiazoline-6-sulfonic acid)) (74 mM stock solution). The laccase were purified from *Sclerotinia sclerotiorum* according to the protocol described [25]. The sample concentrations in the assay mixture were 400, 200, 100, 10 μg/ml for the MeOH extract and 20 μg/ml for the isolated compounds. The content of the generated ABTS^**●+**^ radical was measured at 420 nm after 230 s reaction time and was converted to gallic acid equivalents by the use of a calibration curve (Pearson’s correlation coefficient: *r* = 0.998) constructed with 0, 4, 10, 14, 28, 56, 84 μM gallic acid standards rather than Trolox [22,23]. Experiments were done in triplicate.

### Hemolytic assay

Whole blood (10 ml) from a healthy man was collected into a conical tube containing heparin as an anticoagulant (blood group O was used). Authorization for the collection of blood was obtained from the Medical and Ethical Committee (in Yaoundé-Cameroon). The written informed consent for participation in the study was obtained from a parent of 39 years old. Erythrocytes were harvested by centrifugation for 10 min at 1,000 × *g* and room temperature and washed three times in PBS solution. The top layer (plasma) and the next, milky layer (buffy coat with a layer of platelets on top of it) were then carefully aspirated and discarded. The cell pellet was resuspended in 10 ml of PBS solution and mixed by gentle aspiration with a Pasteur pipette. This cell suspension was used immediately.

For the normal human red blood cells, which are in suspension, the cytotoxicity was evaluated as previously described [26]. MeOH extract **(**at concentrations ranging from 64 to 2048 μg/ml) and compounds **2**–**5** (32 to 512 μg/ml), were incubated with an equal volume of 1% human red blood cells in phosphate buffered saline (10 mM PBS, pH 7.4) at 37°C for 1 h. Ampicillin and chloramphenicol were tested simultaneously. Non-hemolytic and 100% hemolytic controls were the buffer alone and the buffer containing 1% Triton X-100, respectively. Cell lysis was monitored by measuring the release of hemoglobin at 595 nm with a spectrophotometer (Thermo Scientific, USA). Percent hemolysis was calculated as follows:

$$ \frac{\left[\left(\mathrm{A}595\mathrm{of}\ \mathrm{sample}\ \mathrm{treated}\ \mathrm{with}\ \mathrm{compound}\hbox{--} \mathrm{A}595\ \mathrm{of}\ \mathrm{sample}\ \mathrm{treated}\ \mathrm{with}\ \mathrm{buffer}\right)\right]}{\left[\left(\mathrm{A}595\ \mathrm{of}\ \mathrm{sample}\ \mathrm{treated}\ \mathrm{with}\ \mathrm{Triton} \times \hbox{--} 100\ \hbox{--} \mathrm{A}595\mathrm{of}\ \mathrm{sample}\ \mathrm{treated}\ \mathrm{with}\ \mathrm{buffer}\right)\right]}\times 100 $$

### Statistical analysis

Statistical analysis was carried out using Statistical Package for Social Science (SPSS, version 12.0). The experimental results were expressed as the mean ± Standard Deviation (SD). Group comparisons were performed using One Way ANOVA followed by Waller-Duncan Post Hoc test. A p value of 0.05 was considered statistically significant.

## Results and discussion

### Chemical analysis

**Figure 1:**
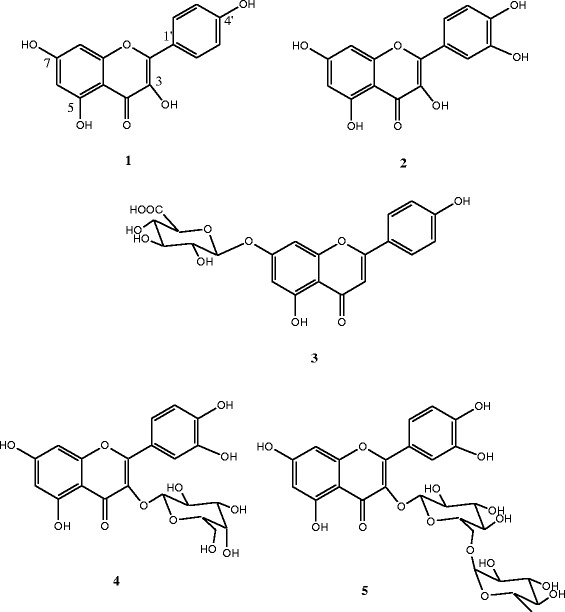
Chemical structures of the isolated compounds from *O. spinosa*. **1**: kaempferol; **2**: quercetin; **3**: apigenin-7-O-β-D-glucuronopyranoside; **4**: quercetin 3-O-β-D-galactopyranoside; **5**: quercetin 3-O-α-L-rhamnopyranosyl (1 → 6) β-D-glucopyranoside.

The MeOH extract of *O. spinosa* leaves was separated by silica gel column chromatography to give several sub-fractions which were further purified by open column chromatography to afford five known compounds previously described in the literature (Figure 1). They included kaempferol (**1**) [15], quercetin (**2**) [16], apigenin-7-O-β-D-glucuronopyranoside (**3**) [17,18], quercetin 3-O-β-D-galactopyranoside (**4**) [16] and quercetin 3-O-α-L-rhamnopyranosyl (1 → 6) β-D-glucopyranoside (**5**) [19].

### Antimicrobial activity

The MeOH extract and four of its isolated compounds (**2**–**5**) were examined *in vitro* against bacterial and fungal species and the results are depicted in Table 1. Kaempferol (**1**), obtained in small amount, was not tested. The MeOH extract and compounds **2, 3, 4, 5** showed selective activities; their inhibitory effects being noted respectively on 7/7 (100%), 7/7 (100%), 4/7 (57.14%), 7/7 (100%) and 4/7 (57.14%) of the studied microorganisms. *Klebsiella pneumoniae* ATCC11296 and *Enterobacter aerogenes ATCC13048* were the most sensitive bacteria while the most sensitive fungi were *Candida parapsilosis* and *Cryptococcus neoformans* IP 90526. The MeOH extract showed only fungistatic activity against yeast strains while the killing effects of many tested samples could be expected on the sensitive strains at the MMC values not more than twofold their corresponding MICs [27]. The MeOH extract**,** compounds **2** and **4** were found to be active against all the microbial strains. Compound **5** was more active than **4** and the later than **2,** against all the bacterial strains. The reverse observations were noted with the fungal strains with compound **2** more active than **4**, and compound **5** being inactive. The three compounds have the same aglycon moiety. Therefore, the sugar moieties at position 3 in **4** and **5** should be responsible for the difference in the observed activity. The lowest MIC value for these tried compounds (8 μg/ml) was recorded with compound **5** on *K. pneumoniae* ATCC11296. This compound displayed the largest antibacterial activity. The antibacterial and antifungal activities of the tried samples were in some cases equal or more important than those of two reference drugs chloramphenicol (MIC = 16 – 64 μg/ml) and nystatin (MIC = 128 – 256 μg/ml), highlighting their good antimicrobial potency. Taking into account the medical importance of the tested microorganisms, this result can be considered as promising in the perspective of new antimicrobial drugs development. The present study showed antimicrobial activity of flavonoids (phenolic compounds) and MeOH extract from *O. spinosa* leaves against the microorganisms including bacterial and fungal species. Compounds **1**–**5** were previously obtained from other sources, but they are isolated here for the first time from the genus *Oncoba*. In addition, this is the first time that secondary metabolites are isolated from *O. spinosa*.

**Table 1:** **Inhibition parameters (**μ**g/ml) of the MeOH extract and compounds from** ***O. spinosa*** **against microbial species**

**Microorganisms**	**Inhibition parameters**	**MeOH extract**	**2**	**3**	**4**	**5**	Reference drugs*
*Enterobacter aerogenes ATCC13048*	MIC	256	64	128	32	32	64
	MBC	512	64	128	64	32	64
	MBC/MIC	2	1	1	2	1	1
*Escherichia coli ATCC8739*	MIC	512	128	>256	128	64	64
	MBC	1024	256	nd	128	64	64
	MBC/MIC	2	2	nd	1	1	1
*Klebsiella pneumoniae ATCC11296*	MIC	256	64	256	32	8	16
	MBC	512	64	256	64	16	16
	MBC/MIC	2	1	1	2	2	1
*Staphylococcus aureus*	MIC	1024	256	>256	128	64	64
	MBC	1024	>256	nd	128	128	64
	MBC/MIC	1	nd	nd	1	2	1
*Candida parapsilosis*	MIC	1024	64	64	128	>256	256
	MFC	>2048	64	128	256	nd	256
	MFC/MIC	nd	1	2	2	nd	1
*Candida albicans* ATCC 9002	MIC	2048	128	>256	256	>256	128
	MFC	>2048	128	nd	256	nd	128
	MFC/MIC	nd	1	nd	1	nd	1
*Cryptococcus neoformans* IP 90526	MIC	1024	64	128	128	>256	128
	MFC	>2048	64	128	128	nd	256
	MFC/MIC	nd	1	1	1	nd	2

*: nystatin for fungi and chloramphenicol for bacteria; nd: not determined; MIC: Minimum Inhibitory Concentration; MBC: Minimum Bactericidal Concentration; MFC: Minimum Fungicidal Concentration.

Flavonoids and their glycosides have attracted considerable interest because of a large variety of biological activities, such as antioxidant [28], antiplasmodial [29], cytotoxic [30], anti-inflammatory [31], antidiabetic [32] and antimicrobial [19,33]. However, no study has been reported on the antimicrobial activity of the compounds **2**–**5** and MeOH extract from the leaves of *O. spinosa* against these types of pathogenic strains. The mechanism of the active compounds (**2**–**5**) is still to be studied; nevertheless, their activity is probably due to their ability to complex with extracellular and soluble proteins and to complex with bacterial cell walls. More lipophilic flavonoids may also disrupt microbial membranes [34].

### Antioxidant activity

**Figure 2:**
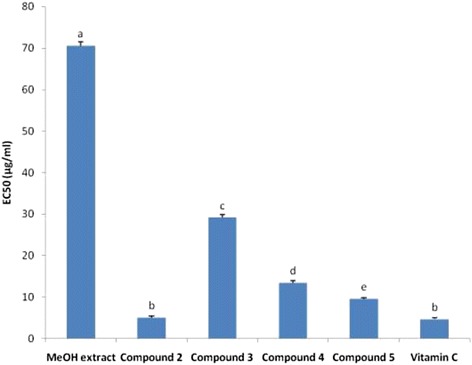
Equivalent concentrations of test samples scavenging 50% of DPPH radical (EC_50_). Bars represent the mean ± SD of three independent experiments carried out in triplicate. Letters a-e indicate significant differences between samples according to one way ANOVA and Waller Duncan test; p < 0.05.

**Figure 3:**
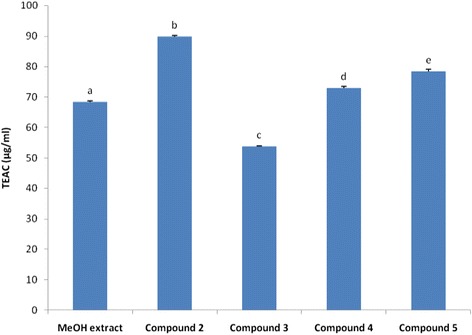
Gallic acid equivalent antioxidant capacity (TEAC; μg/ml) of tested samples. Bars represent the mean ± SD of three independent experiments carried out in triplicate. Letters a-e indicate significant differences between samples according to one way ANOVA and Waller Duncan test; p < 0.05.

Both with DPPH and TEAC methods, compound **2** (EC_50_ = 5.08 μg/mL; TEAC = 89.86 μg/ml) showed the highest antioxidant activity (AOA) followed in decreasing order by compound **5** (EC_50_ = 9.63 μg/ml; TEAC = 78.35 μg/ml), compound **4** (EC_50_ = 13.44 μg/ml; TEAC = 72.95 μg/ml), compound **3** (EC_50_ = 29.16 μg/ml; TEAC = 53.76 μg/ml) and MeOH extract (EC_50_ = 70.56 μg/ml; TEAC = 68.32 μg/ml) (Figures 2 and 3). The free radical scavenger activity of compound **2** is comparable to that of vitamin C used as reference antioxidant drug (EC_50_ = 4.72 μg/ml), highlighting its good antioxidant potency.

In addition to the flavonoid compounds, alkaloids, tannins, sterols, and anthraquinones were previously detected in the 70% aqueous ethanol, hexane and chloroform extracts from *O. spinosa* [6]. Phenolic compounds such as flavonoids are known to be potential antioxidant due to their ability to scavenge free radicals and active oxygen species such as singlet oxygen, superoxide anion radical and hydroxyl radicals [35,36]. Therefore, the presence of such compounds could be responsible for the antioxidant activity found in the MeOH extract. To the best of our knowledge, this is the first systematic screening for the antioxidant activity of the MeOH extract and compounds from *O. spinosa*.

### Hemolytic activity

Human red blood cells provide a handy tool for toxicity studies of compounds, because they are readily available, their membrane properties are well known, and their lysis is easy to monitor by measuring the release of hemoglobin [26]. To investigate the potential use of MeOH extract and compounds **2**–**5**, the cellular toxicity also has to be determined. In this study, none of the tested samples showed hemolytic activities against human red blood cells at concentrations up to 512 μg/ml and 2048 μg/ml for isolated compounds and MeOH extract respectively (results not shown). This finding highlights the fact that the observed biological efficacy is not due to cytotoxicity.

## Conclusion

The phytochemical study of the MeOH extract of *O. spinosa* leaves afforded five known flavonoids including kaempferol (**1**), quercetin (**2**), apigenin-7-O-β-D-glucuronopyranoside (**3**), quercetin 3-O-β-D-galactopyranoside (**4**) and quercetin 3-O-α-L-rhamnopyranosyl (1 → 6) β-D-glucopyranoside (**5**). The MeOH extract and compounds **2**–**5** possess significant antimicrobial and antioxidant activities with no toxicity to human red blood cells. They may be used as phytomedicines at low cost and easily affordable by the target population with caution of clinical studies currently going on in our Laboratory.
